# Efficacy and safety of compound Qingdai enema in rapidly alleviating inflammatory activity in ulcerative colitis: a prospective study

**DOI:** 10.3389/fphar.2026.1776775

**Published:** 2026-06-10

**Authors:** Yao Huang, DanPing Qin, Qiang Yang, Xinyan Yang, YiWen Fang, SiHui Zheng, ChunLi Zhang, Shan Liu

**Affiliations:** 1 The First School of Clinical Medicine, Zhejiang Chinese Medical University, Hangzhou, China; 2 Department of Gastroenterology, The First Affiliated Hospital of Zhejiang Chinese Medical University (Zhejiang Provincial Hospital of Traditional Chinese Medicine), Hangzhou, China; 3 Department of Gastroenterology, Hangzhou TCM Hospital Affiliated to Zhejiang Chinese Medical University, Hangzhou, China; 4 Department of General Medicine, The First Affiliated Hospital of Zhejiang Chinese Medical University (Zhejiang Provincial Hospital of Traditional Chinese Medicine), Hangzhou, China; 5 Medical Department for Senior Cadres, The Third Affiliated Hospital of Zhejiang Chinese Medical University (Zhongshan Hospital of Zhejiang Province), Hangzhou, China; 6 Department of Gastroenterology, Yuyao Traditional Chinese Medicine Hospital, Ningbo, China; 7 The First Affiliated Hospital of Zhejiang Chinese Medical University (Zhejiang Provincial Hospital of Traditional Chinese Medicine), Hangzhou, China; 8 Clinical Evaluation Center, The First Affiliated Hospital of Zhejiang Chinese Medical University (Zhejiang Provincial Hospital of Traditional Chinese Medicine), Hangzhou, China

**Keywords:** compound Qingdai enema, rapid relief, rectal enema, tight junction proteins, ulcerative colitis

## Abstract

**Aim of the Study:**

To observe the efficacy and safety of CQE in rapidly alleviating inflammatory activity in UC.

**Materials and Methods:**

CQE extracts were analyzed by UHPLC–Q Exactive Orbitrap–HRMS. This study used a self-controlled design before and after treatment. A total of 114 UC patients were included and treated with traditional rectal enema administration of CQE. Among them, 107 patients completed the treatment, and 101 patients with complete clinical and laboratory data were included in the final analysis. The primary clinical intestinal symptoms, mucosal endoscopic and pathological changes, laboratory indicators, quality of life scores, and adverse reactions were compared before and after 14 days of enema treatment. A historical comparison was made with previous studies. The expression of intestinal tight junction proteins in the colons of UC patients during active periods was detected using Quantitative Real-time PCR (RT-qPCR) and Western blot (WB).

**Results:**

The main compounds in CQE were identified, including Gallic acid, Matrine, Oxysophocarpine, Ellagic acid, Prim-O-glucosylcimifugin, Cytisinicline, Danshensu, Protocatechuic acid, Cimifugin, Salvianolic acid A Cytisinicline, Indigo, Indirubin, Tryptanthrin and others. The primary endpoint, defined as the change in modified Mayo score, showed a significant improvement after 14 days of CQE treatment (*P* < 0.001). The clinical response rate was 94.06% (95/101), and the clinical remission rate was 66.34% (67/101). The endoscopic mucosal healing rate was 76.24% (77/101), and the histological remission rate was 38.61% (39/101). Compared to before treatment, post-treatment scores for clinical intestinal symptoms, Mayo endoscopic scores (MES), Geboes index, fecal calprotectin (FC), and fecal occult blood test (OB) were significantly reduced (*P* < 0.001). The UCEIS score for the distal rectum and sigmoid colon showed more improvement than the proximal colon (*P* < 0.01). Hemoglobin (HGB), albumin (ALB), and quality of life scores increased (*P* < 0.05), while liver and kidney function indicators showed no abnormal elevations after treatment (*P* < 0.05). Compared to historical data from previous studies using steroids and biologics, CQE significantly improved clinical and endoscopic remission rates. RT-qPCR and WB results demonstrated that the herbal enema helps repair the intestinal mucosal barrier in UC patients.

**Conclusion:**

CQE can rapidly alleviate inflammatory activity in UC within 14 days and improve patients’ quality of life, with a low incidence of adverse events.

## Graphical Abstract

**Figure:**
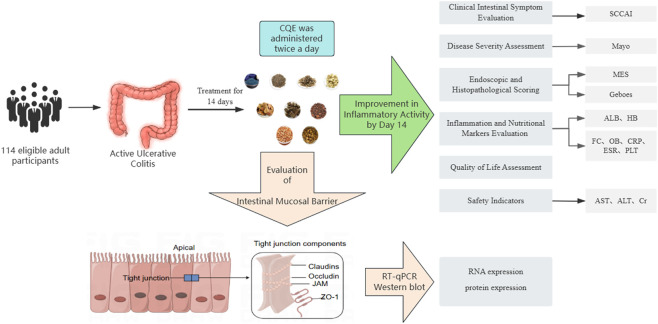

## 1 Introduction

Ulcerative colitis (UC) is a chronic inflammatory disease of the colon, characterized by recurrent episodes of inflammation and remission, with mucosal inflammation typically starting from the rectum and extending to the proximal segments of the colon. UC’s inflammatory activity manifests through symptoms such as diarrhea, mucus or bloody pus in the stool, and abdominal pain, which cause significant discomfort to patients. Therefore, quickly alleviating inflammatory symptoms and improving patients’ wellbeing and compliance is of great importance (Raine et al., 2022). Epidemiological studies in the early 21st century suggest that the incidence of UC in Asia may be rapidly increasing, particularly in China, where the incidence rate has risen sharply in the past decade (Ng et al., 2017).

To reduce disease activity and maintain stable remission, it is essential to find a new treatment method that is rapid, effective, and safe for UC. In China, TCM has been widely used to treat UC. Researches indicate that from 1981 to 2000, over 20% of UC patients used simple Chinese medicine treatment, while 59.1% adopted a combination of TCM and Western medicine (Jiang and Cui, 2002). Common TCM treatment methods include oral and local administration, with rectal enemas and suppositories being the primary means of local treatment, and rectal enemas playing a significant role in UC treatment (Chen et al., 2005). Currently, most evaluations of the efficacy of TCM enemas in treating UC are conducted over extended periods, usually after 1 month or even 6 months of treatment. Few clinical studies focus on the rapid relief of inflammatory activity, hindering the assessment of whether TCM enemas can achieve quick symptom relief. Therefore, research aimed at rapid relief of inflammatory activity is urgently needed. Additionally, many clinical studies on TCM treatment for UC involve the combination of TCM with other medications, making it difficult to independently assess the efficacy of TCM. CQE, developed from long-term clinical practice, is a TCM formula composed of 12 herbs, including Indigo Naturalis (Qingdai powder), Portulaca oleracea (Ma Chi Xian), Patrinia scabiosaefolia (Bai Jiang Cao), and Sophora flavescens (Ku Shen). Our previous research demonstrated that CQE can rapidly improve clinical symptoms in patients with UC. Most patients receiving CQE showed marked improvements in stool frequency, rectal bleeding, and abdominal pain within 1–2 weeks. Moreover, achieving therapeutic targets within a 2-week course significantly enhanced patient adherence, better reflecting the concept of rapid symptom relief (Wetwittayakhlang et al., 2021). Therefore, a 14-day period was selected based on both clinical practice and pharmacodynamic considerations and determined to be appropriate to evaluate the rapid anti-inflammatory and symptom-relieving effects of CQE. In addition, there is a significant correlation between achieving clinical remission (based on symptoms/score indicators) within 2 weeks and improvement in endoscopic/tissue conditions within 8 weeks (Solitano et al., 2025; Chen et al., 2024). A 14-day evaluation window can effectively reflect early efficacy while minimizing the influence of spontaneous remission or other confounding factors. Meanwhile, this relatively short observation period ensures safety monitoring and avoids long-term confounders from concurrent medications or dietary factors (Loftus et al., 2023). This study, with a 14-day treatment period, aimed to evaluate the efficacy of CQE in rapidly alleviating UC inflammation. The results indicate that CQE provides rapid relief of inflammatory activity in UC and achieves remission without steroid intervention.

## 2 Materials and methods

### 2.1 Study design

This was a single-center, single-arm clinical study with Objective Performance Criteria (OPC), employing a prospective self-controlled design before and after treatment. Patients were recruited from the Department of Gastroenterology at the First Affiliated Hospital of Zhejiang Chinese Medical University between May 2019 and December 2023. The study was conducted in accordance with the Declaration of Helsinki and was approved by the Ethics Committee of the First Affiliated Hospital of Zhejiang Chinese Medical University (No. 2018-ZX-025-01). The study was also registered on the International Traditional Medicine Clinical Trial Registry platform (http://itmctr.ccebtcm.org.cn/, NO.: ITMCTR2025000610). Written informed consent was obtained from all participants prior to enrollment, and written consent was also obtained for the collection of clinical samples.

The study was designed in collaboration between the sponsor (the First Affiliated Hospital of Zhejiang Chinese Medical University) and gastroenterologists. Data management and statistical analysis were conducted by a third-party institution. The sponsor collected the data, monitored the study’s progress, and coordinated the manuscript preparation with all authors. All authors had access to the study data and reviewed and approved the final manuscript.

### 2.2 Study population

Based on the Montreal classification, the disease extent was classified into ulcerative proctitis (E1), left-sided UC (E2), and extensive UC (E3) (Satsangi et al., 2006); The severity was categorized as mild, moderate, or severe according to the modified Truelove and Witts classification (Truelove and Witts, 1955). Patients were also categorized by clinical type as initial onset or chronic relapsing (Magro et al., 2017).

#### 2.2.1 Inclusion criteria

① According to the 2017 ECCO Consensus on the Diagnosis and Management of Inflammatory Bowel Disease, the patient was diagnosed with active ulcerative colitis (Magro et al., 2017); ② Age between 18 and 80 years at the time of informed consent; ③ Diagnosed as extensive UC through colonoscopy and other diagnostic tests; ④ Diagnosis of the TCM syndrome of damp-heat in the large intestine, with symptoms such as diarrhea, mucus, or bloody pus in the stool, abdominal pain, tenesmus, a burning sensation in the anus, bloating, dark urine, dry mouth, and bitter taste. TCM signs included a red tongue, yellow greasy coating, and slippery pulse; ⑤ Signed informed consent.

#### 2.2.2 Exclusion criteria

① Inability to undergo colonoscopy or difficulty tolerating enema administration; ② Presence of serious comorbidities such as hematological, cardiovascular, hepatic, renal, or neurological diseases or psychological disorders; ③ Complications such as toxic megacolon; ④ Participation in another clinical studies within the last three months; ⑤ Unstable or severe disease requiring urgent medical intervention; ⑥ Documented allergy or hypersensitivity to any component of the CQE preparation; ⑦ Abnormal liver or renal function at baseline; ⑧ Concurrent use of systemic corticosteroids, immunosuppressants, or biologic agents during the study period.

#### 2.2.3 Dropout criteria

① Use of medications or interventions that could potentially influence study outcomes or interfere with the assigned treatment protocol; ② Failure to complete the prescribed treatment course or alteration of the treatment regimen without authorization; ③ Development of severe complications, new comorbidities, or significant physiological changes rendering continued participation unsafe or inappropriate; ④ Inability to attend scheduled follow-up visits or loss to follow-up, regardless of whether formal withdrawal was declared.

#### 2.2.4 Safety assessment

An *a priori* safety assessment of the CQE formulation was conducted based on existing pharmacological and clinical literature. Known adverse effects, potential herb–drug interactions, and contraindications of each botanical component were reviewed prior to study initiation. All patients were closely monitored for adverse events throughout the intervention, and routine laboratory parameters, including liver and renal function, were assessed before and after treatment.

### 2.3 Sample size

The sample size was calculated based on the primary endpoint, defined as the change in modified Mayo score. Based on the sample size calculation formula: n = [σ(Z1-α/2 + Z1-β)/(μ-μ0)]^2^, and using preliminary trial results, the primary outcome measure was the modified Mayo score, with a pre-treatment score of (8.20 ± 3.27) and a post-treatment score of (6.20 ± 3.90). With α = 0.05 and β = 0.1, it was calculated that 40 patients were needed. Considering a 20% dropout rate, the study aimed to recruit at least 48 patients. In this prospective single-arm study, all eligible consecutive patients during the study period were included to enhance the statistical precision and generalizability of the findings. As a result, a total of 114 patients were ultimately enrolled, exceeding the minimum required sample size. The additional outcomes assessed in this study were considered secondary endpoints and were not used for sample size determination.

### 2.4 Study medication and preparation

CQE consisted of 12 Chinese herbal medicine: 3 g of Qing Dai powder, 30 g of Ma Chi Xian, 15 g of Bai Jiang Cao, 15 g of Ku Shen, 10 g of Shi Liu Pi, 6 g of Er Cha, 30 g of Di Yu, 10 g of Dan Shen, 10 g of Fang Feng, 6 g of Wu Bei Zi, 9 g of Sheng Ma and 15 g of Wu Mei. The botanical origins of all herbs are shown below (Table 1). The CQE was prepared and quality controlled by the Pharmacy Center of the First Affiliated Hospital of Zhejiang Chinese Medical University in accordance with standardized hospital decoction procedures and the Chinese Pharmacopoeia. All herbal materials were weighed according to the original prescription ratio, soaked in water at a 10-fold ratio for 30 min, and decocted twice (30 min for the first extraction and 20 min for the second extraction). The extracts were combined, filtered, and concentrated under reduced pressure to yield a final preparation equivalent to approximately 0.49 g/mL of crude drug. The decoction was administered orally at a dose of 200 mL per administration, twice daily. This preparation procedure was used exclusively for clinical administration and is distinct from the sample preparation method employed for LC–MS analysis.

**TABLE 1:** Components of the CQE, the plant name has been checked with http://www.theplantlist.org.

Chinese name	Full name	Part used	Weight (g)
Qing Dai	*Indigo naturalis*	Foliage	3
Ma Chi Xian	*Portulaca oleracea L*	Herb	30
Bai Jiang Cao	*Patrinia scabra Bunge*	Herb	15
Ku Shen	*Sophora flavescens Aiton*	Root	15
Shi Liu Pi	*Punica granatum L*	Pericarp	30
Er Cha	*Acacia catechu (L.f.) Willd*	Branch	6
Di Yu	*Sanguisorba officinalis L*	Root	30
Dan Shen	*Salvia miltiorrhiza Bunge*	Root	10
Fang Feng	*Saposhnikovia divaricata (Turcz.) Schischk*	Root	9
Wu Bei Zi	*Rhus chinensis Mill*	Leaf	6
Sheng Ma	*Actaea cimicifuga L*	Root	9
Wu Mei	*Prunus mume (Siebold) Siebold & Zucc*	Kernel	15

All botanical drugs used in the CQE preparation are officially listed in the Chinese Pharmacopoeia and are not derived from endangered or protected species. The raw materials were sourced from certified suppliers in compliance with national regulations on medicinal plant use. None of the ingredients are classified as restricted substances, and all materials were obtained through ethical and sustainable channels.

### 2.5 UHPLC–Q exactive orbitrap–HRMS analysis

The herbs of the CQE were mixed in accordance with the original prescription dosage ratios. All twelve herbs were soaked in deionized water in proportion for 1 hour. Subsequently, water equal to ten times the weight of the ingredients was added, and the mixture was boiled for an additional 30 min. After decoction and filtration, the filtrate was concentrated to a 1.0 g/mL density, which was used as the stock solution for analysis. The sample was mixed and subjected to ultrasonic extraction in an ice-water bath, followed by low-temperature precipitation and centrifugation. The resulting supernatant was collected, spiked with an internal standard, and transferred to vials for subsequent analysis. The preparation procedure described for LC-MS analysis was performed solely for chemical profiling.

The metabolomic data analysis was performed by Shanghai Luming biological technology co., LTD (Shanghai, China). An ACQUITY UPLC I-Class plus (Waters Corporation, Milford, USA) fitted with Q-Exactive HF mass spectrometer equipped with heated electrospray ionization (ESI) source (Thermo Fisher Scientific, Waltham, MA, USA) was used to analyze the metabolic profiling in both ESI positive and ESI negative ion modes. Chromatographic separation was performed on an ACQUITY UPLC HSS T3 column (1.8 μm, 2.1 × 100 mm) using a water–acetonitrile mobile phase with gradient elution. The flow rate was 0.35 mL/min and column temperature was 45 °C. All the samples were kept at 4 °C during the analysis. The injection volume was 2 μL. Mass spectrometry data were acquired over a scan range of m/z 100–1,500 with data-dependent MS/MS acquisition.

### 2.6 Data preprocessing and statistical analysis

The original LC-MS data were processed by software XCMS V4.5.1 for baseline filtering, peak identification, integral, retention time correction, peak alignment, and normalization. Main parameters of 5 ppm precursor tolerance, 20 ppm product tolerance. The extracted features were subsequently matched against an in-house database (LuMet-TCM, LuMet Biotechnology Co., Ltd., Shanghai, China), which has been utilized in previous LC–MS-based analyses of herbal medicines (Lu et al., 2025; Pan et al., 2024), and the publicly available HERB database (http://tcmspw.com/herb.php) for compound annotation. Compound identification was based on accurate mass-to-charge ratios (m/z), MS/MS fragmentation patterns, and isotopic distribution.

Compounds were tentatively identified based on XCMS-processed features, and only those with a total matching score greater than 40 were retained for further analysis. After merging and weighting the substances in the positive and negative ion mode, the total content of the relative peak area of the metabolites is set at 100% to obtain a qualitative and quantitative result data matrix, the matrix contains all the information extracted from the original data that can be used for analysis, and subsequent analysis is based on it. Draw an EIC diagram and an MS2 mirror comparison diagram with secondary fragment structure annotations for each identified traditional Chinese medicine ingredient. Draw a pie chart based on the quantity of all identified traditional Chinese medicine components under each chemical classification category.

### 2.7 Administration method

For patients with active UC, treatment was administered via rectal enema without the combination of other medications, using CQE for 14 consecutive days. During enema administration, patients were placed in a left lateral position with their hips elevated. After lubricating the enema tube with paraffin oil, the tube was inserted 15 cm into the rectum. A 50 mL syringe with a soft tube was used to inject the enema solution into the rectum, followed by a 20 mL saline flush. The enema was administered twice a day, once in the morning and once in the evening, with the evening enema retained overnight.

### 2.8 Efficacy evaluation methods

The primary endpoint of this study was defined as the change in modified Mayo score after treatment. Secondary endpoints included clinical symptom scores (SCCAI), endoscopic scores (MES), histopathological scores (Geboes), inflammatory biomarkers (FC, OB, CRP, ESR, WBC, PLT), nutritional indicators (HGB, ALB), quality of life (IBDQ).

#### 2.8.1 Clinical intestinal symptom evaluation

Clinical symptoms (Bowel frequency (day and night), Blood in stool, Urgency of defecation, General wellbeing, Extracolonic features) will be assessed using the Simple Clinical Colitis Activity Index (SCCAI) a validated and widely used clinical index for the assessment of disease activity in UC (Walmsley et al., 1998; Kishi et al., 2022). The total SCCAI score ranges from 0 to 19, with higher scores indicating greater disease activity. Patients’ clinical intestinal symptoms were recorded daily. The SCCAI was assessed at baseline and at predefined follow-up visits (day 4 and day 14) to evaluate changes in clinical disease activity. A SCCAI score of <2.5 was predefined as the threshold for clinical symptom remission (Higgins et al., 2005).

#### 2.8.2 Disease severity assessment

Disease Severity Scoring: Disease severity was scored using the modified Mayo Score for UC before and after enema treatment. Scores were based on the patient’s daily bowel movements, rectal bleeding, colonoscopic findings, and the physician’s overall assessment. The scoring was conducted by a UC specialist physician. Due to the single-arm design, blinding to treatment status was not feasible. However, clinical data were recorded prospectively, and scoring was performed using predefined criteria by experienced clinicians to reduce subjective bias. Mild disease activity was defined as a score between 3-5, moderate activity as 6–10, and severe activity as 11–12 (D'Haens et al., 2007).

Disease Severity Improvement Assessment: Clinical remission was defined as a Mayo score ≤2 with no individual item scoring >1. Clinical response was defined as a ≥30% reduction from baseline Mayo score and a decrease of ≥3 points, with a reduction of ≥1 point in rectal bleeding or a rectal bleeding score of 0 or 1. Clinical remission rate (%) = (number of cases in clinical remission/total cases) × 100%. Clinical response rate (%) = (number of clinically effective cases/total cases) × 100%.

#### 2.8.3 Endoscopic and histopathological scoring

Endoscopic Scoring: Mayo endoscopic scores (MES) were recorded before and after treatment, with scoring based on the most severely affected part of the colon as observed through colonoscopy. The MES is widely used for the evaluation of mucosal inflammation and disease activity in UC. Recent studies have further validated its reliability and clinical utility in assessing treatment response (Khanna et al., 2022; Xu et al., 2022). Endoscopic scoring was performed by experienced endoscopists. Blinding to treatment time point was not implemented; however, evaluations were conducted according to standardized scoring criteria, and consistency was maintained across assessments to minimize inter-observer variability. Mucosal healing was defined as an MES score of 0 or 1. Mucosal healing rate (%) = (number of cases with mucosal healing/total cases) × 100% (Schroeder et al., 1987).

Histopathological Scoring: Biopsy samples were taken from the most severely affected areas before and after treatment. If a patient showed improvement, biopsy sites remained consistent pre- and post-treatment. A single pathologist evaluated the mucosal samples according to the Geboes scoring system. The pathologist was not blinded to the treatment time point; however, all samples were assessed using predefined histological criteria, and evaluations were performed in a consistent manner to reduce subjective bias. Histological remission was defined as a Geboes score <2. Histological remission rate (%) = (number of cases with histological remission/total cases) × 100% (Geboes et al., 2000).

#### 2.8.4 Stratified analysis

By Disease Severity: Patients were grouped into mild, moderate, and severe categories based on disease severity scores, and their improvement, as well as endoscopic scores, were analyzed.

By Disease Extent: Patients were grouped based on disease extent into E1, E2, and E3 categories according to the Montreal classification. Disease severity improvement and endoscopic scores were analyzed for each group.

#### 2.8.5 Inflammation and nutritional markers evaluation

Patients underwent laboratory tests before and after treatment.

Inflammatory Markers: White blood cell count (WBC), platelet count (PLT), C-reactive protein (CRP), erythrocyte sedimentation rate (ESR), FC, and OB. The scoring method for OB was as follows: OB (−) = 0 point, OB (1+) = 1 point, OB (2+) = 2 points, OB (3+) = 3 points.

Nutritional Markers: HGB and ALB.

#### 2.8.6 Quality of life assessment

Patients’ quality of life was assessed using the Inflammatory Bowel Disease Questionnaire (IBDQ), which includes sections on intestinal symptoms, systemic symptoms, emotional function, and social function. A higher score indicated a better quality of life (Guyatt et al., 1989).

#### 2.8.7 Safety indicators

Patients were monitored for safety indicators before and after treatment. These included alanine aminotransferase (ALT), aspartate aminotransferase (AST), and serum creatinine (Scr).

### 2.9 Evaluation of intestinal mucosal barrier status

Collect colon tissue samples from patients before and after treatment. In addition, a control group was established from patients with no abnormalities detected during colonoscopy. All patients have signed informed consent forms.

RT-PCR was used to detect the RNA expression levels of tight junction (TJ) proteins (including JAM, claudin, occludin, ZO-1) before and after treatment to assess the repair of the intestinal mucosal barrier in UC patients. The primer sequences used for RT-PCR are listed in the table (Table 2).

**TABLE 2:** Primer sequences of mRNA.

Genes	Primer sequence
h-β-actin	Forward 5′-CTC​CAT​CCT​GGC​CTC​GCT​GT
	Reverse 3′-GCT​GTC​ACC​TTC​ACC​GTT​CC
h-ZO-1	Forward 5′-GCA​TGA​TGA​TCG​TCT​GTC​CTA​CCT​G
	Reverse 3′-CCG​CCT​TCT​GTG​TCT​GTG​TCT​TC
h-JAM-A	Forward 5′-AGC​TGG​TCT​TTG​ATC​CCC​TG
	Reverse 3′-CCA​TAC​CCA​TTC​CGT​GCC​TC
h-Claudin-1	Forward 5′-CTC​CTA​TGC​GGG​TGA​CAA​CA
	Reverse 3′-GAG​ACC​ACC​ATT​AGG​GCT​CG
h-Occludin	Forward 5′-AGC​AGC​GGT​GGT​AAC​TTT​GA
	Reverse 3′-CCT​CCA​GCT​CAT​CAC​AGG​AC

WB was also be used to detect TJ proteins in patients before and after treatment to evaluate the repair of the UC intestinal mucosal barrier. The antibodies and dilution ratios used were: JAM-A (Abcam, USA, 1:1000); claudin-1 (Abcam, USA, 1:5000); occludin (CST, USA, 1:1000); ZO-1 (Protein tech, China, 1:5000); GAPDH (Affinity, China, 1:5000).

### 2.10 Statistical analysis

Statistical analysis was performed using SPSS 19.0 software. In the descriptive analysis, quantitative data that follow a normal or approximately normal distribution were described using mean ± standard deviation ( $\bar{x}\pm s$ ), while data that do not follow a normal or approximately normal distribution were described using the median (interquartile range), i.e., M (Q). Qualitative data were described using rates (%). For quantitative data that follow a normal or approximately normal distribution, a paired t-test was used, and for data that do not follow a normal or approximately normal distribution, the rank-sum test was used. Categorical data were analyzed using the chi-square test ( x ^2^ test). All statistical tests were two-tailed, and a P-value of less than 0.05 was considered statistically significant.

Data were expressed as the arithmetic mean ± standard error of the mean (SEM). Data analysis was performed using GraphPad Prism 5.0 (GraphPad, USA). One-way analysis of variance (ANOVA) was used to assess statistically significant differences between groups, and Tukey’s test was used for multiple comparisons. A P-value of less than 0.05 indicated a statistically significant difference.

## 3 Results

### 3.1 Qualitative analysis of CQE

**FIGURE 1:**
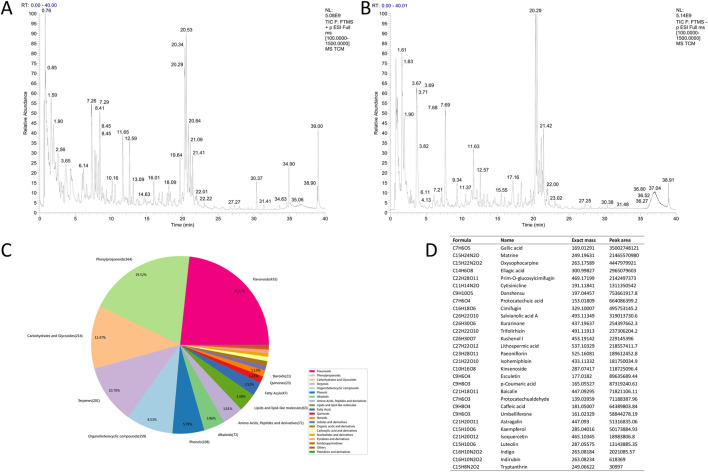
Chemical identification of CQE using UHPLC–Q Exactive Orbitrap–HRMS. **(A)** The chemical total ion chromatogram (TIC) chromatogram of CQE in the positive ion mode. **(B)** The TIC chromatogram of CQE in the negative ion mode. **(C)** Structural classification of compounds contained in CQE. **(D)** Representative chemical components of CQE identified by UHPLC–Q Exactive Orbitrap–HRMS.

CQE extracts were analyzed by UHPLC–Q Exactive Orbitrap–HRMS. The total ion chromatograms in both negative and positive ion models are displayed in Figures 1A,B. A total of over 1,200 compounds in CQE were identified, including flavonoids, phenylpropanoids, carbohydrates and glycosides, terpenes, organoheterocyclic compounds, Phenols, Alkaloids, Amino Acids, Peptides and derivatives, among others Figure 1C. The top compounds with relatively large peak area included Gallic acid, Matrine, Oxysophocarpine, Ellagic acid, Prim-O-glucosylcimifugin, Cytisinicline, Indigo, Indirubin, Tryptanthrin, and others Figure 1D.

### 3.2 Baseline characteristics

The study enrolled 114 patients with UC, and the clinical baseline data are summarized in Table 3.

**TABLE 3:** Basic data of 114 patients with UC( $\bar{x}\pm s$ ,n = 114).

Group		Number
Sex,n (%)	Male	73 (64.04)
	Female	41 (35.96)
Age $\bar{x}\pm s$		46.45 ± 13.52
Course of disease, M(Q)		3.00 (6.75)
Disease extension, n (%)	E1	29 (25.44)
	E2	49 (42.98)
	E3	36 (31.58)
Severity of Illness, n (%)	Mild	42 (36.84)
	Moderate	55 (48.25)
	Severe	17 (14.91)
Disease type, n (%)	Initial onset type	13 (11.40)
	Chronic recurrent type	101 (88.60)
Past drug use, n (%)	5-Asa	79 (66.39)
	Steroids	16 (13.45)
	Immunosuppressants	4 (3.36)
	Biologics	6 (5.04)
	Other	14 (11.76)

### 3.3 Case completion status

**FIGURE 2:**
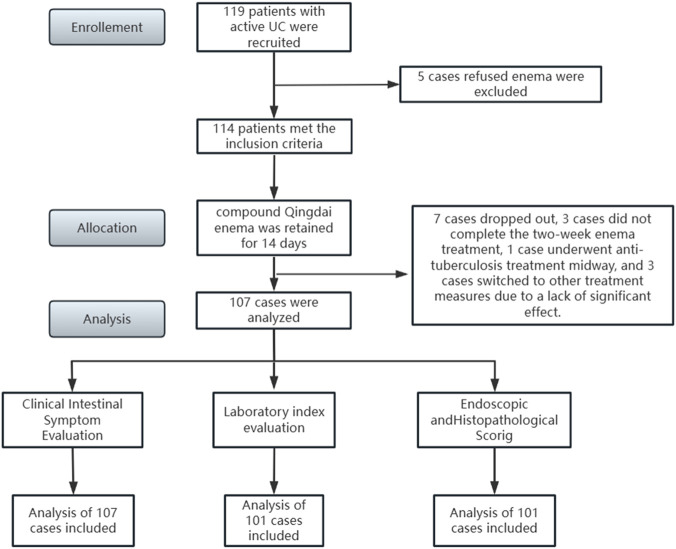
CONSORT flowchart. Note: Missing data in laboratory and endoscopic evaluations were due to incomplete follow-up examinations.

A total of 114 UC patients were enrolled in this study, among which 107 completed the clinical symptom assessment, 101 completed the laboratory index evaluation, and 101 completed the endoscopic and histopathological scoring (Figure 2).

### 3.4 Main results: improvement in inflammatory activity by day 14

#### 3.4.1 Primary endpoint: change in modified Mayo score

The primary endpoint, defined as the change in modified Mayo score, showed a significant improvement after treatment. 101 UC patients underwent modified Mayo scoring before and after treatment. The post-treatment score (2.70 ± 2.30) significantly decreased from the pre-treatment score (7.48 ± 2.44) (*t* = 14.24, *P* < 0.01). According to the defined criteria, all 101 patients showed improvement in disease severity, with a clinical response rate of 94.06% (95/101) and a clinical remission rate of 66.34% (67/101).

#### 3.4.2 Secondary endpoint: clinical symptom assessment (SCCAI)

**FIGURE 3:**
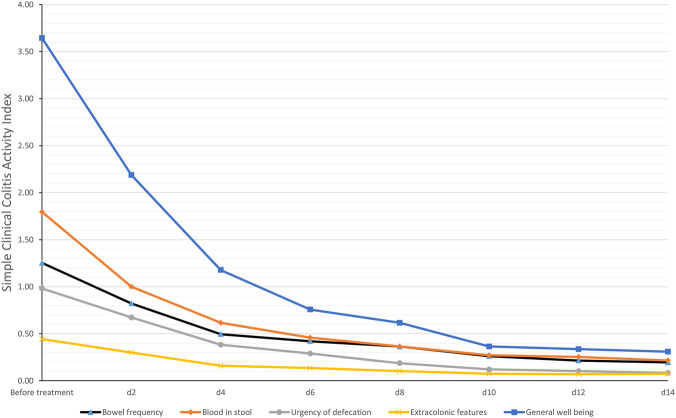
Simple clinical colitis activity index of 107 patients with UC after treatment ( ± ).

By recording patients’ daily symptoms, significant relief was observed after 3 days of enema treatment, with a rapid drop in symptom scores by the 4th day (Figure 3). By Day 14, scores stabilized, reaching or approaching a minimum of 0. Compared with baseline scores for bowel frequency (1.25 ± 0.92), blood in stool (1.79 ± 1.02), urgency of defecation (0.98 ± 0.98), extracolonic features (0.44 ± 0.44), and general wellbeing (3.64 ± 3.07), post-treatment scores were significantly reduced to 0.20 ± 0.54, 0.21 ± 0.61, 0.08 ± 0.28, 0.07 ± 0.20, and 0.31 ± 1.16, respectively (*P* < 0.001).

#### 3.4.3 Endoscopic and histopathological evaluation

**FIGURE 4:**
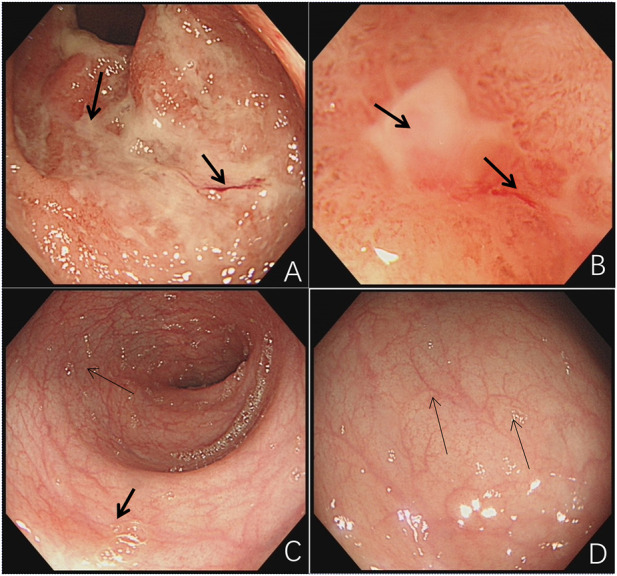
Endoscopic Findings of UC Patients Before and After 14 Days of Enema Treatment. **(A,B)** (Before enema): Rectal mucosa shows congestion, edema with erosion and ulcers, covered with white exudate, loss of vascular pattern, and spontaneous bleeding observed (see **↓**). **(C,D)** (After enema): Rectal mucosa appears smooth, with a mostly restored vascular pattern (see **↓**), and a few small patches of congestion (see ↓).

**FIGURE 5:**
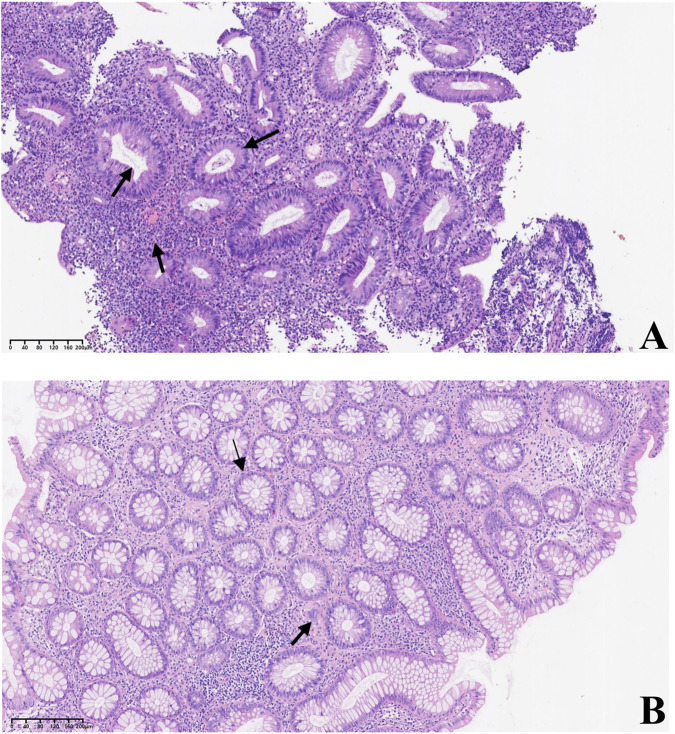
Pathological Findings of UC Patients Before and After 14 Days of Enema Treatment (HE, ×10). **(A)** (Before enema): Crypt abscesses and extensive inflammatory cell infiltration are observed (see “**↓**”). **(B)** (After enema): No crypt abscesses are observed, and inflammatory cell infiltration is significantly reduced compared to before (lesion areas marked with “**↓**”, remission areas marked with “↓”). Note: These histopathological changes are consistent with the Geboes scoring results.

101 UC patients underwent a follow-up colonoscopy 14 days after the enema (Figures 4, 5). Post-treatment, there was significant improvement in colonic mucosal inflammation. For patients with lesions localized to the rectum or rectosigmoid region, symptoms such as mucosal hyperemia and edema diminished, vascular patterns became clearer, and erosion, ulcers, and bleeding improved. In patients with left-sided or extensive colitis, distal rectal mucosa also showed improvement, with mild hyperemia or erosion persisting, but no ulcers or bleeding. The MES score post-treatment (1.06 ± 0.71) was significantly lower than pre-treatment (2.50 ± 0.56) (*t* = 16.06, *P* < 0.05). The distribution of MES score post-treatment showed that 2.97% (3/101) scored 3, 20.79% (21/101) scored 2, 56.44% (57/101) scored 1, and 19.80% (20/101) scored 0, resulting in a mucosal healing rate of 76.24% (77/101).

Post-treatment, inflammation cell infiltration in the mucosal layer of the affected intestinal tissue was significantly reduced. Histopathological findings showed a Geboes score of 2.33 ± 1.36 post-treatment compared to 3.80 ± 1.50 pre-treatment (*t* = 7.27, *P* < 0.001). Based on defined criteria, the histological remission rate was 38.61% (39/101).

#### 3.4.4 Stratified evaluation

##### 3.4.4.1 Disease severity stratification

Among 101 UC patients, based on the modified Mayo score, 24 patients were categorized as mild, 61 as moderate, and 16 as severe. It was found that in mild patients, the post-treatment modified Mayo score (1.29 ± 0.68) significantly decreased from the pre-treatment score (4.38 ± 0.81) (*t* = 3.53, *P* < 0.001); moderate patients had a post-treatment score of (2.54 ± 1.83) compared to (7.66 ± 1.25) pre-treatment (*t* = 6.67, *P* < 0.001); and severe patients had a post-treatment score of (5.44 ± 3.06) compared to (11.44 ± 0.50) pre-treatment (*t* = 8.57, *P* < 0.001). Based on this, clinical remission and efficacy were evaluated for changes in disease severity before and after treatment (Table 2).

Pre- and post-treatment MES scores were assessed, revealing that mild patients had a post-treatment MES score of (0.80 ± 0.67) compared to (2.04 ± 0.45) pre-treatment; moderate patients had (1.05 ± 0.66) post-treatment *versus* (2.56 ± 0.53) pre-treatment; and severe patients had (1.50 ± 0.71) post-treatment *versus* (3.00 ± 0.00) pre-treatment. This was used to evaluate the endoscopic mucosal healing status (Table 4).

**TABLE 4:** Stratified evaluation of disease severity in UC patients.

Disease severity	Cases (n)	Clinical response	Clinical remission	Endoscopic mucosal healing
Mild	24	24/24 (100.00)	17/24 (70.83)	21/24 (87.50)
Moderate	61	59/61 (96.72)	29/61 (47.54)	48/61 (78.68)
Severe	16	12/16 (75.00)	1/16 (6.25)	8/16 (50.00)
x ^*2*^	​	12.691	26.094	7.963
*P*	​	0.002	<0.001	0.019

Data are presented as n (%). Comparisons among groups were performed using the chi-square test. P values indicate differences among mild, moderate, and severe groups.

The data above show that the response to improvement in disease severity is related to the different degrees of the disease, with mild to moderate patients more likely to achieve better improvement responses compared to severe patients. The endoscopic MES also demonstrates the same trend, indicating that mild to moderate patients are more likely to achieve mucosal healing.

##### 3.4.4.2 Lesion extent stratification

In the study of 101 patients with UC, 26 patients were classified as type E1 before treatment, 43 as type E2, and 32 as type E3. It was found that the modified Mayo score for type E1 patients after treatment was significantly reduced to (1.88 ± 1.15) from (6.50 ± 2.29) before treatment. For type E2 patients, the score after treatment was (2.28 ± 1.82), significantly lower than the pre-treatment score of (7.30 ± 2.21). Type E3 patients had a post-treatment score of (3.94 ± 2.98), also significantly lower than the pre-treatment score of (8.50 ± 2.46). Based on this, the degree of improvement in the condition before and after treatment was assessed in two categories: clinical remission and clinical response (Table 5).

**TABLE 5:** Stratified evaluation of lesion extent in UC patients.

Lesion extent	Cases (n)	Clinical response	Clinical remission	Endoscopic mucosal healing
E1	24	24/24 (100.00)	17/24 (70.83)	21/24 (87.50)
E2	61	59/61 (96.72)	29/61 (47.54)	48/61 (78.68)
E3	16	12/16 (75.00)	1/16 (6.25)	8/16 (50.00)
x ^*2*^	​	12.691	26.094	7.963
*P*	​	0.002	<0.001	0.019

Data are presented as n (%). Comparisons among groups were performed using the chi-square test. P values indicate differences among E1, E2, and E3 groups.

The MES was evaluated before and after treatment. It was found that for type E1 patients, the MES after treatment was significantly reduced to (0.65 ± 0.48) from (2.31 ± 0.46) before treatment. For type E2 patients, the post-treatment score was (1.05 ± 0.80), lower than the pre-treatment score of (2.47 ± 0.54). For type E3 patients, the MES after treatment was (1.42 ± 0.67), also significantly reduced from the pre-treatment score of (2.72 ± 0.57). Based on this, the condition of endoscopic mucosal healing was evaluated (Table 5).

The data above indicate that the response to improvement in disease severity is related to the different extents of lesion involvement. Patients with E1 and E2 types are more likely to achieve better improvement responses compared to E3 type patients. Endoscopic MES show that E1 type patients are more likely to achieve mucosal healing compared to E3 type patients, but there is no significant difference in mucosal healing between E2 and E3 type patients.

#### 3.4.5 Evaluation of inflammatory and nutritional indicators

In 101 patients, a panel of inflammatory and disease activity-related biomarkers was evaluated (Table 6). These included FC, OB, CRP, ESR, WBC, PLT, as well as nutritional indicators including HGB and ALB. After 14 days of treatment, only baseline levels of FC and OB were significantly elevated before treatment. After 14 days of treatment, FC levels decreased significantly from (752.95 ± 750.27 μg/g) to (200.89 ± 445.80 μg/g) (*P* < 0.001). OB levels also significantly decreased from (1.46 ± 1.07) to (0.41 ± 0.78) after treatment (*P* < 0.001). Baseline values of blood HGB and ALB were within the normal range, and both indicators showed significant increases after 14 days of treatment (*P* < 0.05, *P* < 0.01). There were no statistically significant differences in WBC, PLT, ESR, or CRP before and after treatment (*P* > 0.05).

**TABLE 6:** Comparison of laboratory parameters at baseline and post-treatment (mean ± SD, n = 101).

Parameter	Baseline	Post-treatment
FC (μg/g)	752.95 ± 750.27	200.89 ± 445.80^***^
OB score	1.46 ± 1.07	0.41 ± 0.78^***^
WBC (×10^9^/L)	6.71 ± 2.22	6.70 ± 1.96
PLT (×10^9^/L)	237.49 ± 83.60	243.58 ± 76.22
ESR (mm/h)	8.44 ± 11.14	7.87 ± 9.68
CRP (mg/L)	5.11 ± 10.80	3.45 ± 6.84
HGB (g/L)	134.28 ± 19.36	137.88 ± 19.20
ALB (g/L)	39.76 ± 5.10	41.95 ± 5.12^**^

Compared with baseline, *P < 0.05, **P < 0.01, ***P < 0.001.

### 3.5 Comparison of the IBDQ scores before and after treatment

In 42 UC patients, quality of life was assessed using the IBDQ. The quality-of-life scores improved significantly after treatment (174.07 ± 26.40) compared to before treatment (107.64 ± 23.85) (*t* = −16.80, *P* < 0.01).

### 3.6 Safety evaluation

Safety was assessed through laboratory parameters and clinical monitoring. No adverse events related to the study treatment were reported, and no treatment discontinuations due to adverse events occurred (Table 7). In terms of laboratory safety, liver and renal function indicators remained stable (Table 8). After 14 days of treatment, liver and kidney function indicators in 101 patients showed no abnormal increases (*P* > 0.05). ALT levels were (16.58 ± 9.22) U/L before treatment and (18.66 ± 10.84) U/L after treatment. AST levels were (17.68 ± 5.85) U/L before treatment and (19.95 ± 7.39) U/L after treatment. Cr levels were (69.58 ± 17.37) μmol/L before treatment and (69.63 ± 18.87) μmol/L after treatment.

**TABLE 7:** Summary of adverse events (n = 101).

Category	n (%)
Any adverse event	0 (0%)
Grade ≥3 adverse events	0 (0%)
Discontinuation due to AE	0 (0%)

**TABLE 8:** Comparison of safety-related laboratory parameters at baseline and post-treatment (mean ± SD, n = 101).

Parameter	Baseline	Post-treatment
ALT (U/L)	16.58 ± 9.22	18.66 ± 10.84
AST (U/L)	17.68 ± 5.85	19.95 ± 7.39
Cr (μmol/L)	69.58 ± 17.37	69.63 ± 18.87

## 4 Evaluation of intestinal mucosal barrier

**FIGURE 6:**
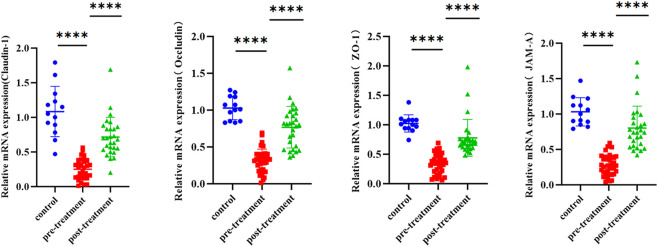
RT-qPCR detection of TJ protein RNA expression in the colon of UC patients. Data are presented as the mean ± SD. The sample sizes were as follows: control group (n = 13), active UC group (n = 35), and post-treatment group (n = 28). Compared with post-treatment groups, *P < 0.05,**P < 0.01,***P < 0.001,****P < 0.0001.

RT-qPCR results show (Figure 6) that RNA expression levels of JAM-A, claudin-1, occludin, and ZO-1 in the colon of patients with active UC are significantly lower than those in the control group (*P* < 0.0001). After 14 days of enema treatment, RNA expression levels of JAM-A, claudin-1, occludin, and ZO-1 increased (*P* < 0.0001).

**FIGURE 7:**
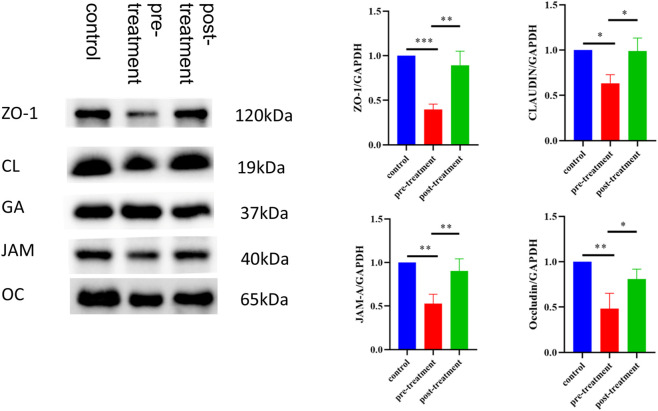
WB detection of TJ protein expression in the colon of UC patients. Data are presented as the mean ± SD Compared with post-treatment groups, *P < 0.05,**P < 0.01,***P < 0.001.

WB results show (Figure 7) that the protein expression levels of JAM-A, claudin-1, occludin, and ZO-1 in the colon of patients with active UC are significantly lower than those in the control group (*P* < 0.001 or *P* < 0.05 or *P* < 0.01). After 14 days of enema treatment, the protein expression levels of JAM-A, ZO-1, and occludin significantly increased (*P* < 0.05 or *P* < 0.01), whereas claudin-1 expression showed no obvious change (*P* > 0.05).

## 5 Discussion

The main goal of UC treatment is to induce and maintain remission. The remission rate for oral mesalazine after 2 months during active UC inflammation is 41.25% (Gong et al., 2012), and steroids are insufficient to achieve mucosal healing. Substituting treatment with azathioprine or biologics carries risks (Sood et al., 2015; Lasa et al., 2022). Each type of medication has limitations in inducing inflammation remission, and steroids cannot be used for maintaining UC remission due to numerous side effects. TCM plays a unique and important role in UC treatment, with local therapies being a key approach. Herbal enema is one of the most commonly used methods, but there has been a lack of focus on how to quickly relieve UC inflammatory activity. This study proposes a TCM strategy for “rapid relief” of UC inflammation using CQE for treatment.

For patients, being able to quickly alleviate clinical symptoms can undoubtedly improve compliance and enhance wellbeing, helping patients to adhere to prescribed guidelines and ultimately achieve treatment goals. In previous studies examining the efficacy of Upadacitinib and Mesalazine, the discussion of “rapid relief” was limited to the alleviation of clinical symptoms only (Loftus et al., 2023; Schreiber et al., 2020). However, in this study, “rapid relief” focuses on symptom improvement and evaluates patients’ clinical conditions daily, with a 14-day endpoint for clinical observation. This comprehensive assessment includes clinical symptoms, endoscopic and histological evaluations, and inflammatory markers such as FC and OB. The therapeutic response achieved within 14 days is considered as rapid relief in this study.

In the present study, the primary endpoint was defined as the change in modified Mayo score, which serves as a composite index reflecting both clinical and endoscopic disease activity in UC. Clinical disease activity was assessed using a combination of the SCCAI, the modified Mayo score, MES and Geboes scores. The SCCAI is a simple, non-invasive, and patient-reported index that is easy to implement in both clinical trials and routine practice, particularly for repeated assessments. It was originally developed as a practical tool applicable in outpatient settings and has demonstrated good correlation with established indices incorporating objective parameters, such as the Seo Index (Kishi et al., 2022). Furthermore, symptom-based indices including SCCAI have been shown to correlate moderately to strongly with endoscopic disease activity, with comparable performance to the partial Mayo score in predicting endoscopic remission (Golovics et al., 2022). In addition, previous studies have highlighted both the widespread use and certain limitations of traditional activity indices, including their reliance on symptoms or complexity in clinical application, underscoring the need for feasible and reproducible tools in routine care (Rapo et al., 2025). Therefore, the combined use of SCCAI with objective indices such as the modified Mayo score and MES in this study enables a more comprehensive and reliable assessment of disease activity. Patients showed significant improvement in clinical intestinal symptoms compared to before treatment (*P* < 0.001). Notably, symptoms began to improve significantly within the first few days of enema treatment. By the fourth day, a sharp improvement in symptoms was observed, demonstrating that CQE has a rapid effect in inducing UC symptom relief. Consistent with the primary endpoint, the modified Mayo score significantly decreased after treatment (*P* < 0.01), with a clinical response rate of 94.06% and a clinical remission rate of 66.34%, suggesting that CQE can quickly and effectively alleviate the inflammatory activity. As patients’ clinical symptoms improved, their quality of life scores also significantly increased after treatment.

The MES and Geboes scores are widely used to evaluate disease activity in UC from endoscopic and histopathological perspectives, respectively (Fluxá et al., 2017). After treatment, both scores significantly decreased (*P* < 0.001), with an endoscopic mucosal healing rate of 76.24% and a histological remission rate of 38.61%. This indicates that treatment can significantly reduce UC inflammation and promote mucosal healing of inflammatory lesions. However, most patients who achieved endoscopic mucosal healing had an MES score of 1. Previous studies have shown that there is a difference in mucosal healing quality between patients with an MES score of one and those with a score of 0, with a score of 0 indicating better healing (Kanazawa et al., 2019). Additionally, the histological remission rate was lower than the endoscopic mucosal healing rate, suggesting that future treatments should focus more on histological remission to achieve high-quality mucosal healing, which will help patients achieve long-term stability after disease remission.

Further analysis was conducted based on disease activity and the extent of the lesions. It was found that patients with mild, moderate, and severe UC all showed significant improvement after treatment, with mild patients achieving a significantly higher clinical remission rate than moderate and severe patients. The clinical response rate for mild and moderate patients was also significantly higher than for severe patients. The MES scores were significantly lower than before treatment. Comparing the three groups, disease activity had a notable impact on mucosal healing, with mild and moderate patients achieving significantly better mucosal healing rates than severe patients.

Patients were categorized into E1, E2, and E3 groups based on the extent of lesions, and it was found that all three groups showed significant improvement after treatment. Although there were no significant differences in clinical remission rates among the three groups, the clinical response rates were significantly higher in E1 and E2 patients compared to E3 patients (*P* < 0.01). The MES scores for all three groups significantly decreased after treatment, with notable differences in the degree of mucosal healing, particularly with E1 patients achieving significantly better mucosal healing than E3 patients. One study found that the risk of severe disease severity in E3 patients is 2.58 times higher than in E1 and E2 patients, indicating that a broader extent of lesions correlates with more severe disease (Zheng et al., 2022). Given the association between submucosal inflammatory activity in UC patients and disease recurrence (Christensen et al., 2017), these findings suggest that after achieving the goal of rapid relief of inflammatory activity, it is crucial to implement subsequent maintenance therapy.

FC and OB can serve as non-invasive indicators for monitoring and evaluating mucosal inflammation (D’Amico et al., 2020). In this study, both significantly decreased after 14 days of enema treatment (*P* < 0.001), indicating that CQE not only improved mucosal inflammation but also reduced the expression of FC and OB. Furthermore, the baseline values of HGB and ALB showed further increases (*P* < 0.05), reflecting an improvement in the nutritional status of the patients after treatment.

Indigo naturalis has previously been associated with safety concerns, particularly when administered orally and at high doses (Xu et al., 2024; Cho et al., 2020). However, in the present study, CQE was administered *via* topical enema at a relatively low dose and for a short duration, which may substantially reduce systemic exposure. Importantly, no serious adverse events were observed during the study period, and no clinically relevant abnormalities in liver or renal function were detected. Nevertheless, given the known safety concerns associated with Indigo naturalis, cautious patient selection, short-term use, and close monitoring remain essential. Further controlled studies with larger sample sizes are warranted to comprehensively evaluate the long-term safety of this formulation.

The quality of mucosal healing is related to intestinal TJ proteins. Therefore, we examined the RNA and protein expression of intestinal TJ proteins in the colons of patients with active UC. It was found that in patients with active UC, TJ protein expression at both the RNA and protein levels was significantly lower compared to the control group (*P* < 0.0001). After 14 days of enema treatment, TJ protein RNA expression increased (*P* < 0.05 or *P* < 0.01), and TJ protein levels also increased (*P* < 0.05 or *P* < 0.01). This indicates that in patients with active UC, the intestinal TJ protein expression is reduced, leading to damage to the intestinal mucosal barrier, but TJ protein expression increases after 14 days of TCM enema. These findings suggest that TCM enema helps repair the intestinal mucosal barrier in UC patients and improves the quality of mucosal healing.

Chemical profiling by UHPLC–Q Exactive Orbitrap–HRMS revealed that CQE contains multiple constituents, including gallic acid, matrine, indigo, and related compounds, which may represent an important material basis for its therapeutic effects. Previous studies have demonstrated that some of these constituents possess anti-inflammatory and intestinal barrier–protective properties. For instance, gallic acid has been reported to alleviate experimental colitis induced by DSS and TNBS, accompanied by reduced inflammatory responses and improved intestinal barrier function, partly through enhancement of TJ protein expression (Zhu et al., 2019; Yu et al., 2023). Matrine and related alkaloids have similarly been shown to ameliorate intestinal inflammation and maintain mucosal homeostasis, partly through preserving TJ protein expression (Jiang et al., 2024). In addition, indigo-containing preparations have been shown to improve epithelial integrity by restoring the distribution and expression of TJ proteins, including ZO-1 and Claudin-1, in models of intestinal injury (Kawaguchi et al., 2023). Although the present study is primarily a clinical investigation and does not aim to elucidate detailed molecular mechanisms, the observed upregulation of TJ–related markers after treatment suggests that these constituents may, at least in part, contribute to the improvement of intestinal barrier function. As this study was a single-arm trial without a control group, several previously published clinical trials of UC therapies, including budesonide and vedolizumab, were reviewed to provide contextual reference. For example, a clinical trial of budesonide foam reported a clinical remission rate of 50.9% and an endoscopic remission rate of 59.1% after 6 weeks of treatment (Naganuma et al., 2016). Similarly, oral budesonide therapy has been associated with a clinical remission rate of approximately 53.98% (Ford et al., 2011). In addition, treatment with vedolizumab has been reported to achieve clinical remission and endoscopic remission rates of 47.1% and 40.9%, respectively (Feagan et al., 2013). Differences in study populations, treatment regimens, endpoints, and study designs should be taken into account when interpreting these findings, and the comparisons provided here are intended for contextual reference only.

Comparisons with standard therapies were based on historical data and should be interpreted cautiously. Nevertheless, the observed rates of clinical and endoscopic remission were comparable to or higher than those reported for steroid-based therapies and biologics in similar patient populations, many of whom had previously shown suboptimal responses to conventional treatments. Baseline evaluations in this study showed that most patients had poor responses to mesalazine (66.39%), immunosuppressants (3.36%), or biologics (5.04%). The results suggest that in cases where most conventional treatments are ineffective, CQE provides an effective response, outperforming immunosuppressants, steroids and biologics, with a shorter onset of action. This suggests that the characteristics of TCM formulas—multiple components, multiple targets, and multi-pathway actions—may overcome the limitations of single large molecule drugs that operate through a single pathway and target and may represent a promising steroid-sparing option for the rapid induction of remission.

Nevertheless, several limitations should be acknowledged. First, this was a single-center prospective study with a relatively modest sample size, which may limit the generalizability of the findings. Second, the study employed a single-arm design without a randomized control group, which limits the ability to establish causal relationships between the intervention and the observed outcomes. Although significant improvements were observed after treatment, these changes may have been influenced by potential confounding factors, placebo effects, or the natural course of the disease. In addition, the absence of blinding in clinical, endoscopic, and histopathological assessments may introduce potential assessment bias, although standardized procedures were applied to mitigate this limitation. In the present study, a controlled design was not feasible due to clinical and ethical considerations, as patients presented with active disease requiring timely treatment, and withholding treatment or assigning placebo was considered inappropriate. Third, the follow-up period was relatively short, and therefore the long-term efficacy and safety of this intervention remain to be further clarified. In addition, although molecular analyses of TJ proteins were performed in the present study, the underlying biological mechanisms were not fully elucidated. Further experimental studies are required to clarify the precise molecular pathways involved. Therefore, the findings of this study should be interpreted with caution. Future multicenter randomized controlled trials with larger cohorts and longer follow-up durations are warranted to validate these findings and further elucidate the mechanisms underlying the therapeutic effects of CQE in UC.

## 6 Conclusion

The results of this study indicate that CQE may rapidly reduce inflammatory activity in UC, significantly improving clinical intestinal symptoms, promoting mucosal healing, and achieving histological remission. CQE treatment was also associated with increased expression of intestinal tight junction proteins, which may contribute to improved mucosal barrier integrity. This aligns with the current trend in UC treatment, shifting from symptom relief to promoting mucosal healing. Additionally, clinical improvement was observed within 14 days in many patients, and steroid-free remission occurred in a proportion of cases. These results suggest that CQE may represent a promising adjunctive therapeutic option for UC.

## Data Availability

The original contributions presented in the study are publicly available. This data can be found here: 10.6084/m9.figshare.32503773.
